# Epidemiological, virological and clinical characterization of a Dengue/Zika outbreak in the Caribbean region of Costa Rica 2017–2018

**DOI:** 10.3389/fcimb.2024.1421744

**Published:** 2024-06-26

**Authors:** Claudio Soto-Garita, Tatiana Murillo, Ileana Chávez-Peraza, Josué Campos-Ávila, Grace Prado-Hidalgo, Jan Felix Drexler, Andres Moreira-Soto, Eugenia Corrales-Aguilar

**Affiliations:** ^1^ Research Center for Tropical Diseases (CIET) and Faculty of Microbiology, University of Costa Rica, San José, Costa Rica; ^2^ National Reference Centre for Virology, Costa Rican Institute for Research and Education on Nutrition and Health (INCIENSA), San José, Costa Rica; ^3^ Siquirres Integral Healthcare Center (CAIS), Costa Rican Social Security Fund (CCSS), Limón, Costa Rica; ^4^ Talamanca Healthcare Center, Costa Rican Social Security Fund (CCSS), Limón, Costa Rica; ^5^ Charité-Universitätsmedizin Berlin, Corporate Member of Freie Universität Berlin, Humboldt-Universität zu Berlin, Institute of Virology, Berlin, Germany; ^6^ German Centre for Infection Research (DZIF), Associated Partner Charité-Universitätsmedizin Berlin, Berlin, Germany

**Keywords:** arboviruses, Dengue, Zika, chikungunya, outbreak, RT-qPCR, phylogenetics

## Abstract

The increase in incidence and geographical expansion of viruses transmitted by the *Aedes* mosquitoes, such as dengue (DENV) and zika (ZIKV) in the Americas, represents a burden for healthcare systems in tropical and subtropical regions. These and other under-detected arboviruses co-circulate in Costa Rica, adding additional complexity to their management due to their shared epidemiological behavior and similarity of symptoms in early stages. Since diagnostics of febrile illness is mostly based on clinical symptoms alone, we gathered acute-phase serum and urine from 399 samples of acute dengue-like cases from two healthcare facilities of Costa Rica, during an outbreak of arboviruses from July 2017 to May 2018, and tested them using molecular and serological methods. The analyses showed that of the clinically presumptive arbovirus cases that were reported, only 39.4% (n=153) of the samples were confirmed positive by RT-PCR to be DENV (DENV (10.3%), CHIKV (0.2%), ZIKV (27.3%), or mixed infections (1.5%). RT-PCR for other alphaviruses and flaviviruses, and PCR for *Leptospira* sp were negative. Furthermore, to assess flavivirus positivity in post-acute patients, the negative sera were tested against Dengue-IgM. 20% of sera were found positive, confounding even more the definitive number of cases, and emphasizing the need of several distinct diagnostic tools for accurate diagnostics. Molecular characterization of the *prM* and *E* genes from isolated viruses revealed that the American/Asian genotype of DENV-2 and the Asian lineage of ZIKV were circulating during this outbreak. Two different clades of DENV-2 American/Asian genotype were identified to co-circulate in the same region and a difference in the platelet and leukocyte count was noted between people infected with each clade, suggesting a putative distinct virulence. Our study sheds light on the necessity for healthcare strategies in managing arbovirus outbreaks, emphasizing the importance of comprehensive molecular and serological diagnostic approaches, as well as molecular characterization. This approach aids in enhancing our understanding of the clinical and epidemiological aspects of arboviral diseases during outbreaks. Our research highlights the need to strengthen training programs for health professionals and the need to increase research-based on laboratory evidence for diagnostic accuracy, guidance, development and implementation of public health interventions and epidemiological surveillance.

## 1 Introduction

Diseases, where the primary symptom is fever, are common in primary healthcare facilities in developing countries (Yactayo et al., 2016; Gutierrez-Barbosa et al., 2020; Pokharel et al., 2020; Srisawat et al., 2022; Mejía et al., 2023). However, a non-specific clinical presentation coupled to the absence of confirmatory laboratory tests in primary healthcare facilities reduces the accuracy of diagnostics (Malavige et al., 2023; Mejía et al., 2023). Epidemiologic links aid to determine the cause of the fever, for instance, in hyperendemic regions for dengue virus (DENV) circulation, this virus is suspected as the most common etiological agent of fever during the rainy season.

DENV is part of a group of viruses transmitted by arthropods like mosquitoes and ticks known as Arboviruses (Guzman and Harris, 2015; Weaver et al., 2018). Arboviruses include flaviviruses such as DENV and Zika (ZIKV), and alphaviruses, for example chikungunya (CHIKV). These viruses are transmitted by *Aedes* mosquitoes having human-to-human transmission cycles in urban areas and zoonotic cycles involving wildlife and arboreal mosquitoes in rural settings (Guzman and Harris, 2015; Wilder-Smith et al., 2017). The reproduction of *Aedes* mosquitoes is favored during the rainy season in tropical and subtropical regions due to the accumulation of water providing suitable settings for larvae to grow (Regis et al., 2014; Rahman et al., 2021). Over the last decades, conditions in tropical and subtropical countries worldwide have favored the emergence and reemergence of arboviral infections due to multiple factors such as climate variability, increased urbanization, human mobility, insecticide resistance, limitations in vector control programs and others (Kyle and Harris, 2008; Guzman and Harris, 2015; de Souza and Weaver, 2024). Only in the Americas, infections caused through the *Aedes* mosquitoes have experienced an unprecedented rise. Dengue cases increased from 1.5 million cumulative cases in the 1980s to 16.2 million in the 2010s, with the four DENV serotypes circulating in the region (San Martín et al., 2010; Dick et al., 2012). Adding to the increase in DENV transmission, the emergence and rapid spread to most countries of the Americas of CHIKV in 2013, and ZIKV in 2015 has posed new challenges for public health authorities, impacting healthcare services, and inflicting a substantial socioeconomic burden (Halstead, 2015; Faria et al., 2017).

The co-circulation of DENV, CHIKV, and ZIKV has presented a challenge for determining the cause of febrile diseases as it is difficult to differentiate these agents epidemiologically and clinically (Waggoner et al., 2016; Ramos-Castañeda et al., 2017). During the first days of the disease, an ample range of signs and symptoms can occur, going from an asymptomatic or oligosymptomatic disease (60–80%), to patients presenting fever, headaches, myalgias or bone pain (DENV or CHIKV), arthralgias (DENV or CHIKV), edema of the extremities (CHIKV and or ZIKV), hemorrhages (DENV or CHIKV), leukopenia, thrombocytopenia, and other unspecific manifestations complicating the clinical diagnose (Yactayo et al., 2016; Guanche Garcell et al., 2020).

Laboratory diagnosis of arboviral diseases is based on the detection of the virus or an immune response to the infection (Guzman and Harris, 2015). Viremia can usually be detected from serum or plasma samples during the first four to six days, and up to seven days in the case of CHIKV. During this acute phase of the disease, direct detection techniques including virus isolation and viral RNA detection by reverse transcription–polymerase chain reaction (RT-PCR) provides a definitive diagnosis (Peeling et al., 2010; Chusri et al., 2014; Waggoner et al., 2016). Urine samples are also useful, especially in detecting ZIKV, since the virus has higher loads and is excreted for a longer time in urine (Gourinat et al., 2015). Once the acute phase is over, approximately from the sixth day after symptoms onset, serology is the method of choice for presumptive diagnosis (Peeling et al., 2010; Chusri et al., 2014; Zhang et al., 2016). However, the detection of virus-specific IgM or IgG antibodies can be challenging, in particular after several or sequential ZIKV and DENV infections, because cross-reactivity can occur due to the high degree of structural and sequence homology between ZIKV and other flaviviruses (Lanciotti et al., 2008; Gourinat et al., 2015; Durbin, 2016). Furthermore, unspecific positive cross-reactions with other infectious diseases have been reported, such as malaria, HIV-1 and COVID-19 (Lam and Devine, 1998; Watt et al., 2000; Vanroye et al., 2021).

In this study we investigated an outbreak of febrile diseases detected between August 2017 to May 2018 in two hyperendemic regions for DENV transmission in Costa Rica. According to the current normative of Costa Rica and the WHO recommendations, patients from geographical areas where DENV transmission has been serologically documented before are diagnosed as DENV infection positive based on clinical characteristics and epidemiological nexus (WHO, 2009; Ramírez et al., 2023). Confirmatory laboratory diagnosis is required only in patients living in areas where previous cases and/or circulation of the *Aedes* mosquito has not been reported or in pregnant women because of the risk of congenital defects caused by ZIKV (Hernández-Chaves et al., 2016; Benavides-Lara et al., 2021). Furthermore, cases of CHIKV and ZIKV have been reported in Costa Rica suggesting not only co-circulation of these viruses but also missing the putative emergence of newly not detected or unknown arboviruses (Ministerio de Saud de Costa Rica and Grupo técnico Nacional de Enfermedades Vectoriales, 2014; Romero-Vega et al., 2023). Both study sites are in the Caribbean coast of Costa Rica, characterized by a tropical climate with a temperature range of 27–30°C, high precipitation and low altitude (de Cambio Climático (MINAE) et al., 2022). These climatological and geographical variables provide the ideal conditions for the replication of arboviruses and their vector mosquitoes, hence the circulation of DENV is endemic in this region which presents almost yearly the highest number of cases in the country (Rahman et al., 2021; Barrantes Murillo et al., 2022; Piche-Ovares et al., 2022). More specifically regarding our study sites, the number of detected arboviral infections in Siquirres during 2017, were 297 of dengue, 435 of zika and 7 of chikungunya; and in 2018, 112 of dengue, 85 of zika and 2 of chikungunya. In Talamanca, the cases of reported arboviral diseases during 2017 were 30 of dengue, 46 of zika and 2 of chikungunya, while in 2018 there were 91 of dengue and 8 of zika (Alfaro-Nájera, 2023). Neighboring countries, Nicaragua and Panamá, have reported circulation of the four DENV serotypes, ZIKV and CHIKV (Gordon et al., 2013; Araúz et al., 2016; Wang et al., 2016; Díaz et al., 2019; Gordon et al., 2019).At least for one of these arboviruses, previous molecular studies from our group have suggested the introduction of DENV from Nicaragua (Soto-Garita et al., 2016).

We characterized the clinical, virological, and epidemiological features of acute dengue-like cases at two primary healthcare facilities in the Caribbean region of Costa Rica and compared the clinical data with laboratory confirmatory RT-qPCR for DENV, ZIKV and CHIKV to determine the cause of the febrile disease and for those negative samples with more than 6 days of symptom onset, DENV IgM was tested. Furthermore, molecular characterization of DENV and ZIKV successfully isolated viruses was performed to elucidate the circulating serotypes and genotypes during this outbreak. Our findings show the co-circulation of these three viruses but most importantly, highlight the high number of cases misdiagnosed or not confirmed and exemplify how difficult the diagnostic of febrile disease has become in arboviral hyperendemic areas. Our results emphasize the need to strengthen continuous training programs for health professionals and the need to increase research based on laboratory evidence that can serve as guidance for the development and implementation of public health interventions and epidemiological surveillance.

## 2 Materials and methods

### 2.1 Study sites, patient selection criteria and sample collection

This study was conducted with samples submitted to the Medical Virology Laboratory at the University of Costa Rica between August 2017 to May 2018 from the Siquirres Integral Healthcare Centre (CAIS) and Talamanca Healthcare Centre of the Costa Rican Social Security Fund (CCSS) both located in the Caribbean region of Costa Rica. Samples from ZIKV, DENV or CHIKV-presumptive diagnosed outpatients were sent for laboratory diagnostic confirmation. A suspected or presumptive arboviral clinical case was defined as a patient residing in these two areas with an acute febrile illness, and at least two or more of the following manifestations: headache, myalgia, arthralgia, rash, hemorrhagic manifestations, or leukopenia. Due to incomplete reporting, retro-orbital pain and vomiting were not included in our analyses. All analyzed samples derived from presumptive and clinically confirmed cases of DENV, ZIKV and/or CHIKV were anonymized before sending to the virology laboratory, and thus the informed consent requirement was waived. Nevertheless, this study was included as part of an ongoing study “Molecular Characterization of Arboviral Infections’’ reviewed and approved by the Ethics Committee of the University of Costa Rica (VI-273–2017) and as part of the diagnostic program of our laboratory (ED-3257). Some clinical features, leukocytes and platelets counts, presumptive diagnosis and origin of the patient were provided when submitted to the laboratory. Two sets of samples were obtained: acute sera were taken at 6 days or less upon symptoms onset, and paired sera and urine (for ZIKV diagnosis) when symptoms onset was longer than 6 days or unknown. All samples for virological diagnostic were stored at 4°C and transported in less than 48 hours to the research laboratory.

### 2.2 Molecular diagnostic tests and laboratory confirmation of presumptive clinical cases

Virus RNA was extracted from 200 μl serum or urine using MagNA Pure LC RNA Isolation Kit I (Roche, Basel, Switzerland) following the manufacturer instructions in a MagNA Pure LC 2.0 extraction system (Roche, Basel, Switzerland). DNA for leptospirosis analyses was extracted from urine samples with a NucleoSpin Tissue DNA extraction kit following the manufacturer instructions (Macherey-Nagel GmbH, Duren, Germany). All RNA and DNA used were quantified and controlled for contamination. Detection and confirmation of DENV, ZIKV and CHIKV was performed in RNA samples using real-time reverse transcription PCR (RT-PCR) with Modular Diagnostic Kits for Dengue, Zika, and Chikungunya viruses and, Multiplex RNA Master Mix in the LightCyclerII (Roche, Basel, Switzerland) following the manufacturer instructions. Furthermore, all negative samples for these three viruses were tested in a pan-flavivirus and a pan-alphavirus PCR protocol previously published (Kuno et al., 1998; Sánchez-Seco et al., 2001), and DNA from urine was tested for leptospirosis using a PCR protocol published by (Harkin et al., 2014). Dengue serotyping was done using the protocol from Lanciotti et al. with the respective serotype controls (Lanciotti et al., 1992).

### 2.3 Dengue IgM detection in RT-PCR negative samples

Since some outpatients might have an unclear or undefined day of symptom onset and thus the RT-PCR resulted negative due to absence of viral nucleic acids in the sample, all DENV-, ZIKV-, and/or CHIKV-negative samples by RT-PCR were tested for Dengue IgM using the Panbio Dengue IgM Capture ELISA (Standard Diagnostics, Seoul, South Korea) following the manufacturer instructions. Positive samples were classified as presumptive positive for Flavivirus IgM. No second serum sample could be requested for seroconversion confirmation.

### 2.4 Statistical analysis

Statistical analysis of the leucocytes and platelets counts was performed with GrapPad Prism v 7.3 using One-Way ANOVA for comparison of leucocyte and platelet count of confirmed diagnosis, and unpaired T Test for comparison of leucocyte and platelet count between the both clades of DENV-2.

### 2.5 Virus isolation of DENV and ZIKV from clinical samples

All serum samples were diluted 1/10 and inoculated in 24 well plates with Vero cells (ATCC CCL-81) with Minimum Essential Medium (MEM) (Sigma-Aldrich, Darmstadt, Germany) and 2% Gibco fetal bovine serum (FBS) (Thermofisher Scientific, Massachusetts, USA) as described previously (Soto-Garita et al., 2016). The cells were first incubated for 1 hour at 37°C with 5% CO_2_ to promote virus adsorption. Then 800 μL of MEM with 2% FBS were added for a final dilution of 1/50. These were incubated approximately for 2 weeks at 37°C with 5% CO_2_. All serum samples were also diluted 1/30 and inoculated in 24 well plates with C6/36 cells (ATCC CRL-1660) in Gibco RPMI medium (Thermofisher Scientific, Massachusetts, USA) with 2% FBS, but if cells were lysed during initial inoculation, additional cells were added (Soto-Garita et al., 2016). C6/36 cells were incubated for approximately 2 weeks at 28°C with 5% CO_2_. During the 2-weeks incubation, supernatants were collected, and confirmatory RT-PCRs were performed to check for viral presence. Positive isolates were then determined through confirmatory RT-PCR and the presence of cytopathic effect for DENV through the presence of syncytial cells and for ZIKV, refringent cells. Viruses were expanded in Vero cells and stocks were frozen at -80°C.

### 2.6 Sequencing of DENV and ZIKV isolates and phylogenetic analysis

Nucleic acid from successfully isolated viruses were extracted using the MagNA Pure LC RNA Isolation Kit I (Roche, Basel, Switzerland) following the manufacturer instructions in a MagNA Pure LC 2.0 extraction system (Roche, Basel, Switzerland) to perform E gene sequencing and phylogenetic analysis. For DENV and ZIKV, cDNA encompassing the *prM* and *E* gen were amplified by PCR as described elsewhere (Díaz et al., 2006; Li et al., 2017). ZIKV *E* gen PCR products were then purified using Exonuclease I and FastAP Thermosensitive Alkaline Phosphatase treatment (Thermo Fisher Scientific, Waltham, MA). Both strands of the amplicons were sequenced using Sanger sequencing (Macrogen Inc., Seoul, South Korea). Previously described E protein gene sequences of geographically related strains of DENV-1 and DENV-2 were obtained from GenBank for analyses. Multiple sequence alignments were performed with MAFFT v7.520 (Katoh and Standley, 2013). Phylogenetic trees were assembled using the maximum likelihood statistical method based on TIM2+F+G4 substitution model by the program IQ-Tree2 v2.3.2 (Kalyaanamoorthy et al., 2017; Minh et al., 2020). The robustness of the resulting tree was established by ultrafast bootstrap analysis with 3 000 replications (Hoang et al., 2018).

## 3 Results

### 3.1 Demographic and clinical characteristics of the analyzed samples

The 399 acute-phase serum and urine samples from clinically presumptive patients for arboviruses were analyzed. The median age of the presumptive cases was 32 years (range: 5–92), 55.5% were from females and 43.7% from males (0.8% did not specify sex). The most frequent age group for presumptive cases was 29–37 years, while patients older than 69 years old represented only 1% of the total samples. 357 samples were from Siquirres while 41 samples were collected in Talamanca. The highest number of samples were collected in Siquirres during August-September 2017 whereas in Talamanca during September-October 2017. In both cases, ZIKV infections were the most prevalent diagnosis during this time frame.

**Figure 1:**
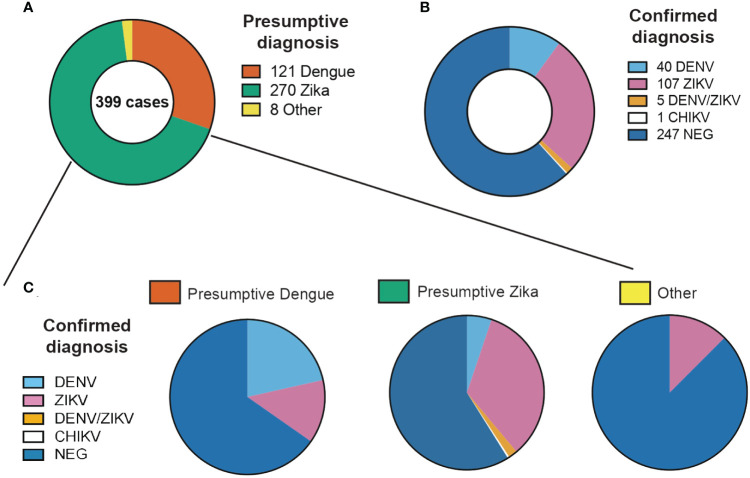
Proportion of clinical cases evaluated in this study classified by **(A)** Presumptive diagnosis from clinical evaluation, **(B)** Confirmed diagnosis from laboratory confirmation and **(C)** Confirmed diagnosis of each of the presumptive diagnosis categories.

According to clinical findings and epidemiological nexus, 265 patients had a presumptive ZIKV-, 106 patients of DENV- and 4 patients of CHIKV-diagnosis. For 23 samples no specific virus was suspected but rather a combination of the three or two of them. Those patients with a presumptive diagnosis of DENV presented fever (98%), headache (75.4%) and arthralgia (68%). Rash (90.5%) was the most prevalent symptom in patients with a presumptive diagnosis of ZIKV (**Figure 1A**). The blood counts of patients with DENV presumptive diagnosis had leukopenia and thrombocytopenia in 44.3% and 47% of the cases, respectively (**Figures 1B, C**). In the case of ZIKV, 17% presented with leukopenia and 4% with thrombocytopenia (**Figures 1B, C**). Statistical comparisons detected significant differences in the leukocyte counts between patients with a presumptive diagnosis of DENV or ZIKV but not among other diagnoses. In the platelet counts, significant differences were detected among the three presumptive diagnoses investigated.

### 3.2 Laboratory confirmation of DENV, ZIKV and CHIKV infection via RT-PCR and presumptive positive serology against flaviviruses

**Figure 2:**
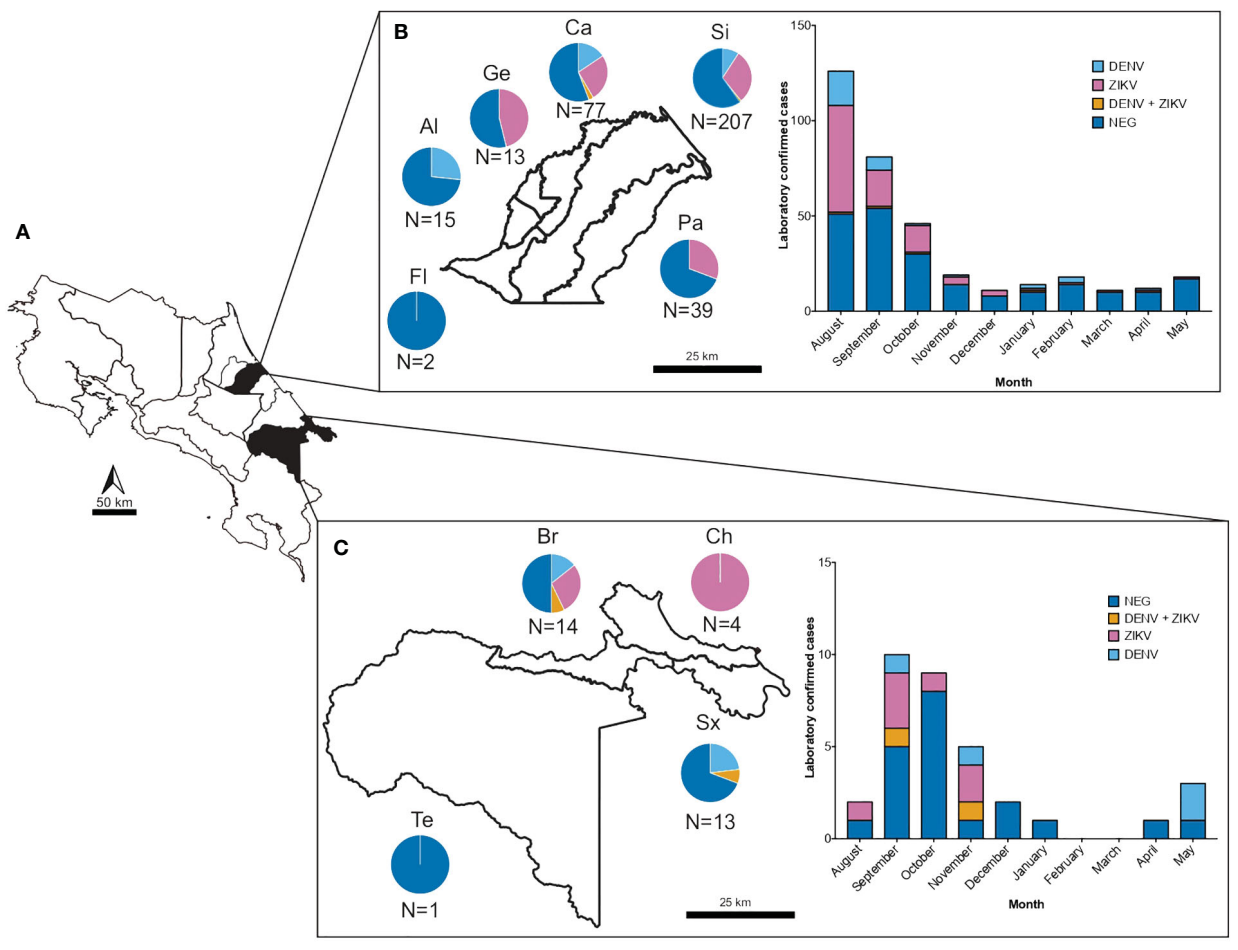
Number of samples and confirmed cases of DENV and ZIKV from suspected arboviral infections collected from August 2017 to May 2018 in two health care services located in dengue hyperendemic regions in Costa Rica. **(A)** Map of Costa Rica depicting in black shading the geographical regions of sample collection. **(B)** Number of samples collected and confirmed cases of DENV (light blue), ZIKV (pink) and DENV-ZIKV coinfections (yellow) in the municipality of Siquirres according to district (Fl: Florida, Al: La Alegría, Ge: Germania, Ca: El Cairo, Si: Siquirres and Pa: Pacuarito) and sampling month. **(C)** Number of samples collected and confirmed cases of DENV (light blue), ZIKV (pink) and DENV-ZIKV coinfections (yellow) in the municipality of Talamanca according to district (Te: Telire, Br: Bratsi, Ch: Cahuita and Sx: Sixaola) and sampling month.

From the 399 samples with a presumptive diagnosis of arboviral infection, only 157 samples (39.4%) were PCR confirmed as having DENV, ZIKV or CHIKV (**Table 1**). 109 (27.3%) were positive for ZIKV, 41 (10.3%) for DENV and 1 (0.2%) for CHIKV. When doing only a clinical differential diagnostic only 35% (92/265) and 23% (24/106) of cases were confirmed in the RT-PCR as ZIKV and DENV respectively. Clearly showing how frequently these arboviral infections are misdiagnosed. Confirmed cases were detected in Siquirres from August 2017 until February 2018 (**Figure 2B**) whereas in Talamanca were only detected from August until November 2017 (**Figure 2C**).

**Table 1:** Laboratory confirmation through real-time RT-PCR of presumptive cases of DENV, ZIKV and CHIKV in serum and urine samples collected between August 2017 until May 2018 in Siquirres and Talamanca.

Patient Data	Total	ZIKV	DENV	DENV+ZIKV	CHIKV
Total: N (% of positive results from total samples)	153 (39.4%)	107 (27.3%)	40(10.3%)	5(1.5%)	1 (0.2%)
% Female % Male	55.5 43.7	52.3 47.7	63.4 36.6	83.3 16.7	0.0 100.0
Median ages, (range)	32 (5–92)	35 (6–74)	36 (6–92)	27 (19–42)	13
Days from onset of symptoms to diagnosis, (range)	3 (1–22)	3 (1–22)	3 (1–8)	2 (1–3)	2
Presumptive diagnosis (confirmed/presumptive) *	157/399 (39%)	92/265 (35%)	24/106 (23%)	6/0	0/4 (0%)

*The confirmed and presumptive diagnosis cases correspond to those cases where laboratory analyses confirmed the initial diagnosis made based on symptoms and epidemiological nexus vs those not confirmed.

The combined analysis of serum and urine samples allowed the detection of 6 patients co-infected with DENV and ZIKV in both municipalities, these patients were between the range of 19–42 years old and 83.3% were female, being two of them pregnant (**Table 1**, **Figure 2**). In these cases, samples were collected between 2 days (range: 1–3) from onset of symptoms (according to the self-report of the patient), and interestingly, 5 patients were positive for ZIKV only in urine samples while only one was positive in urine and serum, showing concomitant viremia for both ZIKV and DENV. The most frequent symptoms present in these patients with coinfections were rash (83.3%), fever (66.7%), headache (50%) and arthralgia (50%), and none required hospitalization. Blood counts showed mild thrombocytopenia (121 x10^3^/µl; reference range: 150–400 x10^3^/µl) only in a male patient, and leukopenia (3.3–3.10 x10^3^/µl; reference range 4–10 x10^3^/µl) in three of the co-infected cases.

The only confirmed positive result for CHIKV was a 17-year-old male with a presumptive diagnosis of ZIKV, he presented himself to the healthcare service with fever, chills, rash, headache, and arthralgias. Blood count analyses showed a mild leukopenia (leukocyte count 3.9 x10^3^/µl) without thrombocytopenia.

RT-PCR negative samples for DENV, ZIKV, and/or CHIKV were further tested in a pan-flavivirus and pan-alphavirus RT-PCR. We aimed to detect any other arboviruses which were not so usually reported in our country or any new emergent arbovirus belonging to these two families. Only amplicons with band sizes other than expected were obtained, however for confirmatory purposes, they were sequenced. No other virus was detected. Thus, other arboviruses apart from DENV, ZIKV and CHIKV were not detected during this outbreak.

Furthermore, we pursued to detect DNA from *Leptospira* sp in urine, since the clinical presentation of leptospirosis can be confounded with Dengue infection, but all samples analyzed resulted negative for *Leptospira* (Brown et al., 2010; Thompson et al., 2015; Loong et al., 2022). Upon profound analysis of patient´s blood counts, since no clinical presentation was suggestive of malaria as a possible cause of the febrile illness, and at that specific time point there were no reports of malaria in these areas of Costa Rica, *Plasmodium* infection was also clinically and epidemiologically ruled out (Ministerio de Salud, 2019).

All RT-PCR negative samples were tested for the presence of Dengue IgM, since the accurate day of fever onset can be missed or can be mistakenly reported by patients. WHO suggests that this kind of serological assay and its results should be considered as presumptive diagnosis and not as confirmation of infection (Katzelnick et al., 2017; Raafat et al., 2019). These assays commonly present false negative results if they are performed too soon after infection and no IgM was yet produced or present false positive results because of antibody cross reactivity with other Flaviviruses or other pathogens (Kerkhof et al., 2020; Musso and Desprès, 2020; da Rocha Queiroz Lima et al., 2021; Stiasny et al., 2021; Chan et al., 2022). From 242 RT-PCR negative samples (minus 8 samples with not enough serum for testing) we obtained 47 Dengue IgM positive results (20%) and 6 inconclusive results (2.5%). A second serum sample should have been asked for to confirm seroconversion by plaque reduction neutralization assays but due to anonymity, this was not feasible. Therefore, we characterized these patients as still having a presumptive DENV-, ZIKV-, or CHIKV-infections and not as a confirmed infection.

### 3.3 Clinical characteristics of the ZIKV and DENV confirmed cases

Of the 109 patients with RT-PCR-confirmed ZIKV diagnosis, 52.3% were females and 47.7% were male (**Table 2**). Their median age was 35 years (range 6–74) and the medium days from onset of symptoms to diagnosis was 3 days (range 1–22). As for risk factors, 2 patients were pregnant but none of them required hospitalization and occurrence of congenital malformations (if present) at birth was not followed upon. According to sample type, the number of ZIKV infections detected were 65 in urine, 24 in serum, and 20 in both urine and serum. Days from symptom onset to diagnosis ranged from 1–22 days (median 3.7) for urine samples, 1–6 days (median 2.21) for serum samples and 1–6 days (median 2.6) for both sample types. The most prevalent symptom was rash, followed by fever, and arthralgia. Abnormalities in either the platelets or leukocytes counts were found in 23.8% of the patients.

**Table 2:** Clinical and laboratory characteristics of patients with PCR-confirmed ZIKV infections.

Presumptive and laboratory confirmed ZIKV cases (Total:N)	Presumptive Diagnosis	Laboratory Confirmed Diagnosis
	265	109
Signs and symptoms		
Fever	78.4%	78.9%
Rash	90.5%	89.0%
Chills	30.2%	30.3%
Headache	50.2%	55.0%
Arthralgia	58.5%	62.4%
Myalgia	37.3%	41.3%
Vomiting	4.9%	3.7%
Abdominal pain	3.0%	2.8%
Diarrhea	1.9%	0.9%
Cough	5.7%	4.6%
Blood count results		
Thrombocytopenia	11 (4.1%)	8 (7.3%)
Leukopenia	45 (16.9%)	18 (16.5%)

In total, 41 patients had a laboratory confirmation of DENV, of which 63.4% were females and 36.6% were male (**Table 3**). The median age of the laboratory confirmed cases was 36 years (range: 6–92) and the medium days from onset of symptoms to diagnosis was 3 days (range: 1–8). None of the diagnosed patients required hospitalization. The most prevalent symptom was fever (92.7%), followed by myalgia and arthralgia (75%), and rash (48.8%). It is noteworthy that 63.4% of the confirmed patients had leukopenia.

**Table 3:** Clinical and laboratory characteristics of patients with PCR-confirmed DENV infections.

Presumptive and laboratory confirmed DENV cases (Total:N)	Presumptive Diagnosis	Laboratory Confirmed Diagnosis
	106	41
Signs and symptoms		
Fever	98.1%	92.7%
Rash	30.2%	48.8%
Chills	61.3%	46.3%
Headache	75.5%	63.4%
Arthralgia	67.9%	75.6%
Myalgia	58.5%	75.6%
Vomiting	21.7%	12.2%
Abdominal pain	12.3%	4.9%
Diarrhea	3.8%	0
Cough	11.3%	0
Blood count results		
Thrombocytopenia	50 (47%)	19 (46.3%)
Leukopenia	47 (44.3%)	26 (63.4%)

**Figure 3:**
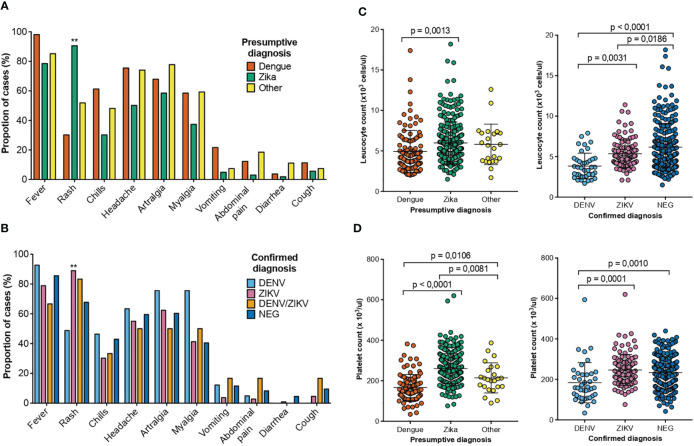
Clinical and laboratory findings of patients with a presumptive/confirmed diagnosis of DENV and ZIKV collected between August 2017 to May 2018 in two health care services from dengue hyperendemic regions of Costa Rica. **(A)** Percentage of patients that presented the clinical symptom or sign for each presumptive diagnosis DENV (orange), ZIKV (green) and other (yellow). **(B)** Percentage of patients that presented the clinical symptom or sign for each confirmed diagnosis of DENV (light blue), ZIKV (pink), DENV/ZIKV (mustard) and negative (blue) **(C)** Total leucocyte counts for the patients with a presumptive diagnosis of DENV (orange), ZIKV (green) and other (yellow) and confirmed diagnosis of DENV (light blue), ZIKV (pink) and negative (blue) **(D)** Total platelet counts for the patients with a presumptive diagnosis of DENV (orange), ZIKV (green) and other (yellow) and confirmed diagnosis of DENV (light blue), ZIKV (pink) and negative (blue) p-values are depicted when significant differences were detected.

The most common symptom during ZIKV infection was a rash while during DENV infection was fever. The difference between DENV and ZIKV causing rash is maintained even if the diagnostics was a presumptive or a confirmatory one (**Figures 3A, B**). DENV infected patients presented more frequently with thrombocytopenia and leukopenia compared to ZIKV infections. But regarding leucocytes and platelets counts, the same difference in parameters is found in presumptive vs confirmed cases in either virus infection (**Figures 3C, D**). Thus, our findings suggest that symptoms and laboratory counts cannot really guide into a confirmation of the virus causing the infection.

### 3.4 Phylogenetic analysis of DENV and ZIKV isolates

**Figure 4:**
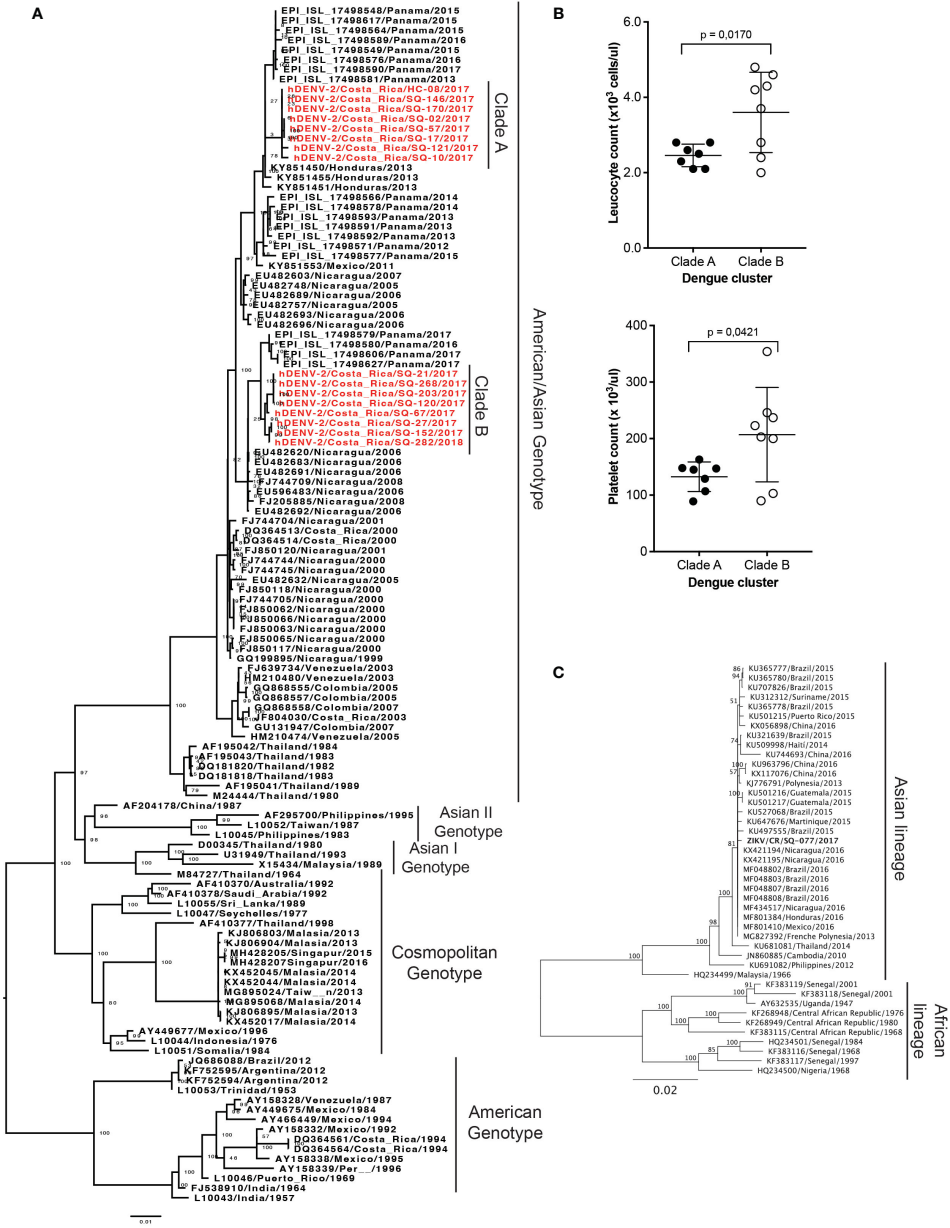
Phylogenetic analysis of DENV and ZIKV isolates obtained from patient samples with RT-PCR-confirmed infections from Siquirres (SQ) or Talamanca (HC). **(A)** Phylogenetic classification of 16 DENV isolates obtained from patient serum samples from Costa Rica in the American/Asian genotype conforming two separate clusters classified as clades A and B, both in red. **(B)** Comparison of leucocyte and platelet counts in the patients from where isolates from clade A (n=8) or B (n=8) were obtained, *p*-values are depicted when significant differences were detected. **(C)** Phylogenetic classification of 1 ZIKV isolate obtained from a patient serum sample from Costa Rica in the Asian lineage. Bootstrap values are depicted.

We obtained 16 DENV isolates from 16 serum samples of patients with RT-PCR-confirmed DENV infections from Siquirres (SQ = 15) and Talamanca (HC = 1), which were sequenced for phylogenetic classification. The analysis grouped all isolates in the American/Asian genotype but in two separate clusters which were then identified as clade A (n=8) and B (n=8) (**Figure 4A**). The percentage of identity between sequences of both clades is around 98%. Both clades are phylogenetically related to isolates from other Central American countries and Mexico. Furthermore, the leucocyte and platelet count of the patients from each clade were compared (**Figure 4B**). Significant differences were obtained in the leukocytes (*p* = 0.017) and platelet (*p* = 0.042) counts between clades A and B, having the patients with infections from Clade A lower counts.

Only one isolate was obtained from the serum of a ZIKV infected patient from Siquirres which was classified in the Asian lineage (**Figure 4C**). This isolate is phylogenetic related to isolates from Nicaragua, Brazil, Martinique, Mexico, Guatemala, and French Polynesia.

## 4 Discussion

We employed molecular diagnostics to examine a febrile disease outbreak in two dengue hyperendemic regions within the Caribbean region from Costa Rica. Our aim was to validate the clinical presumptive diagnosis of arboviral infections, including DENV, ZIKV, or CHIKV, while also to identify certain important epidemiological aspects, isolate viruses, and investigate virus phylogenetics. The results showed that from a total of 399 samples analyzed, only in 157 (39.5%) samples DENV, ZIKV or CHIKV infections were confirmed through a RT-PCR test. From the positive samples, the most prevalent virus detected during this outbreak was ZIKV followed by DENV, while only one case of CHIKV was confirmed. The combined analysis of urine and serum samples increased the detection possibility of ZIKV, since from 109 RT-PCR confirmed ZIKV cases, 91 (83.5%) were positive in RNA extracted from the urine samples. The most common symptom during ZIKV infection was a rash while during DENV infection was fever. DENV infected patients presented more frequently with thrombocytopenia and leukopenia compared to ZIKV infections. Phylogenetic analysis of DENV obtained isolates identified two distinct clades with putative different virulence showing an impact in leucocyte and platelet counts. All DENV isolates are phylogenetically classified as the American/Asian genotype and the one ZIKV isolate was classified in the Asian lineage.

The WHO DENV and ZIKV testing guidelines point out that confirmatory laboratory testing of suspected cases might not be feasible or cost-effective during epidemics or large outbreaks, which is the case for these two study sites investigated. In this context, clinicians at healthcare facilities, diagnose DENV and ZIKV based on the patient clinical presentation and epidemiological nexus. Our results showed that even though sometimes the presumptive clinical diagnosis is correct, in a high percentage of samples the viral infection is not or cannot be confirmed even in hyperendemic arbovirus regions. This might introduce a bias in the WHO-country specific report of numbers of infections and the patient clinical management. Nowadays it is even more difficult since with new viruses causing flu-like illness with fever are widespread, such as SARS-CoV-2, thus confounding the presumptive clinical diagnosis (Joubert et al., 2021; Gérardin et al., 2022; Wong et al., 2023). Nevertheless, the high percentage of asymptomatic presentations for these arboviruses not accounted by clinical diagnosis might balance at the end the official confirmed total cases reported per country. Our aim is not to criticize the WHO guidelines or the national surveillance system but to i) exemplify how difficult the diagnostic of arboviruses has become in hyperendemic areas, ii) highlight the need to strengthen continuous training programs for health professionals, and iii) show the need to increase research based on laboratory evidence that can serve as guidance for the development and implementation of public health interventions and epidemiological surveillance. It is clear the much-needed development and implementation of an easy, fast, affordable, and accurate laboratory test to differentiate among these three arboviruses (Bosch et al., 2017), despite the potential limitations for precise diagnosis posed by the confounding clinical presentation, patients seeking timely medical care, the availability of healthcare providers in an area or country, and/or financial and personnel resources. Although antiviral therapy for these three viral illnesses does not exist yet and most infections are mild, diagnosis is still important to rule out other causes that would have different courses of treatment, to manage severe cases of dengue such as haemorrhagic dengue and shock, and to enhance clinical follow-up on control for children born from ZIKV positive mothers or for other clinical outcomes or sequelae such as Guillain-Barré-Syndrome (Solomon et al., 2000; Brasil et al., 2016; Christie et al., 2023).

In some cases, serology for the detection of virus specific IgM has been implemented to be able to diagnose those patients arriving to the health clinics 6 days after symptom onset (Bozza et al., 2019). In this study, dengue IgMs were detected in 20% of negative RT-PCR samples suggesting arboviral positivity in them. Though a Zika IgM test might have been more appropriate in this case, cross-reactivity has been extensively reported in serological tests against these viruses making this method highly unspecific and difficult to be interpreted (Musso and Desprès, 2020; da Rocha Queiroz Lima et al., 2021; Weiß et al., 2023; Zambrana et al., 2024). A proper dissection of arbovirus serology requires the gold standard for serological definition of specific positive sera, plaque reduction neutralization tests (PRNT). However, during an acute flavivirus infection, a polyclonal activation of antibodies is produced, and cross reactivity is very high (Kerkhof et al., 2020; Musso and Desprès, 2020; Stiasny et al., 2021). Therefore, PRNTs should be used exclusively in sero-epidemiological investigations and with a paired sample in hyperdendemic countries for serological purposes. Alternatively, seroconversion testing of the patients with IgM positive samples against a specific arbovirus would have given a more accurate presumptive diagnosis for infection, but it was not possible in this study. Thus, we think the detection of dengue IgM positive samples should be taken cautiously, as they might indicate arboviral infection or can be the result of cross-reactivity with other pathogens.

Of interest is the high percentage of clinical presumptive DENV, ZIKV or CHIKV infections that were not confirmed through the laboratory tests performed. This might be due to several reasons. First, inadequate sample handling, maintenance, and storage, though this was prevented by sample storage at -4°C and fast transportation to the laboratory. Second, the patient behavior, for example, the day of symptoms onset can be highly inexact due to the unspecific flu-like symptoms presented during these infections, or other unrelated reasons such as symptom onset during weekends, impossibility of sick leave permission to visit the healthcare facility, or even reluctance to seek medical attention, among others. This might affect the moment that patients would visit the healthcare facility in a time corresponding not longer than 6 days after symptom onset, preventing viral detection through RT-PCR. In the case of ZIKV diagnosis, we were able to confirm and show that the concomitant analyses of urine and sera enhances the possibility of detection. This has already been described by many others (Gourinat et al., 2015; De M. Campos et al., 2016; Zhang et al., 2016) and it is highly recommended for ZIKV diagnostics, coupled to the simplicity of urine sample collection and to expand the time frame for diagnostics by RT-PCR more than 6 days after symptoms onset.

Many other infectious diseases, such as leptospirosis, malaria, and even viruses from the same viral families, might be confounded in the clinics with DENV, ZIKV, and/or CHIKV since they share many symptoms. All the patients presented themselves in the clinics with fever and highly unspecific “flu-like symptoms”. Blood analyses also did not show any tendencies such as severe thrombocytopenia suggesting one arbovirus over other. Therefore, we pursued to detect a plethora of different pathogens using an anti-*Flaviviridae* and an anti-*Alphaviridae* semi nested RT-PCRs and sequencing the results. All fragments sequenced did not prompted identification of any other virus. Also, we ruled out malaria since blood analysis did not show any indication for this pathogen and circulation during these years was not reported. Furthermore, we excluded leptospirosis infection in samples that were appropriate for this bacteria diagnostic. This leaves an open question to which other culprits might be causing these unspecific symptoms with a non-severe clinical development to not require hospitalization. Further in-depth analysis of other pathogens of unknown local circulation or novel pathogens should be considered.

It is well known that virus isolation has low sensitivity and viral loads found in the sample do correlate with the success or failure of viral isolation (Jarman et al., 2011; Dangsagul et al., 2021). We pursued to isolate every sample that showed a positive RT-PCR without taking in account Ct values obtained. This might have influenced the low number of isolates that we obtained. Perhaps using a lower serum dilution in comparison to the one that we used for this approach, and taking in account the time passed upon symptom onset might have yielded more isolates from the samples.

The phylogenetic analysis of DENV isolates confirmed the presence of the Asian/American genotype of DENV-2, coinciding with previous findings from our group, though at that time frame (2007–2013) the American genotype was also concomitantly found (Soto-Garita et al., 2016). Although we did not obtain any isolates for this outbreak from other DENV serotypes, genotype V of DENV-1 has been also detected before (Soto-Garita et al., 2016). Phylogenetic analyses of DENV-3 or DENV-4 (detected locally for the first time in 2022) have not yet been performed from Costa Rican isolates. The Asian/American genotype of DENV-2 is the most predominant in the neighboring country of Panamá, and in general in the Latin America continent (Ramos-Castañeda et al., 2017; Díaz et al., 2019). Previous research determined that DENV lineages are shared between Mexico and Central America, which its sensible from a geographical point of view (Ramos-Castañeda et al., 2017). Particularly, in the case of Costa Rica where migration movements between Nicaragua and Costa Rica are common and DENV introductions from Nicaragua have been described before (Soto-Garita et al., 2016). This can be the explanation for the topology of the tree observed in **Figure 4A**, where Nicaraguan isolates from around 2006 and the sequences from this study share a recent common ancestor. Furthermore, Panamanian isolates from around 2017 are closely related to the Costa Rican sequences, suggesting a significant spread of this genetic variant in the region. When assessing evolution relationships between isolates obtained from the two locations of the study, we find that both clades were co-circulating in Siquirres, while Clade A was the only detected in Hone Creek (**Figure 4A**). These findings suggest that Clade A ancestors could have been introduced to Siquirres from circulating viruses in the Southern Caribbean region of Costa Rica. Nevertheless, more sequences are needed to understand the dengue intra-epidemic exchange dynamics within Costa Rica (Hapuarachchi et al., 2016). The same patterns are also appreciated with ZIKV phylogenetic analysis, where Costa Rican sequence clustered near Nicaraguan isolates.

The differences detected in platelet and leucocyte counts between DENV clades suggests that the co-circulation of different DENV genotypes or clades causing infections in the same geographical areas may impact the outcome/severity of disease. Thus, highlighting the importance of laboratory genomic surveillance of arboviruses when outbreaks of severe diseases occur, aiming to identify the possible reasons behind the variations in the severity of disease. However, none of the patients from the samples included in this project required hospitalization obscuring if the detected differences are due to deviations in viral virulence and/or pathogenicity. Nevertheless, it is difficult to determine if the differences observed can really be associated to DENV clades, their interaction with previous DENV or any flavivirus infections, or other reasons at the host or genetic level. Further genomic and virological characterization of these DENV isolates could inform into the putative, if any, virulence mechanisms producing these differences in platelet and leucocyte counts and their possible impact on the disease.

There are several limitations to this study. First, we did this research shortly after ZIKV was introduced to our country (Metsky et al., 2017; Thézé et al., 2018; Gutierrez et al., 2023), therefore enhanced clinical awareness and surveillance were in course. This could explain in the high positivity rate regarding ZIKV infection that we obtained. Since we used pre-existing clinical data, the available information was incomplete, disregarding disease severity, high risk occupational groups and possible co-morbidities. Also, these two areas have been identified as hot spots for diseases by the National Ministry of Health, since they are near international borders, have a lot of immigration and temporal-work national migrating people and particular arbovirus-vector-climate/environmental characteristics (Mayer et al., 2017; Weaver et al., 2018; Messina et al., 2019; Chan et al., 2022; de Souza and Weaver, 2024; Katzelnick et al., 2024). Therefore, it can be possible that the febrile like illness disease might be caused by something else that we did not test for. We did not seek for convalescent sera and, thus, could not perform acute vs. convalescent sera neutralization assays to confirm infections after the pre-determined time window of sample collection for the RT-PCR was overridden. Also, there are significant differences in number of samples between the two areas analyzed. We also did not ask for data such as travel history, which can confound the data obtained. Furthermore, this study was limited to only two areas in Costa Rica, so the data presented and discussed here not necessarily represent the entire country. Nevertheless, the same conclusions are obtained from both sampling sites. Using E gene instead of whole genome for phylogenetic analysis could be considered as also a limitation; though, given the evolutionary pressure in this region, E gene phylogenetics have been used widely with similar results as whole genome approaches (King et al., 2008; Ali and Ali, 2015; Rodríguez-Aguilar et al., 2022).

The emergence of arboviral diseases in other geographical regions so far free from these common arboviruses, has been continuously increasing in the last several years due to climate change. For example, autochthonous cases of Dengue, Zika and/or Chikungunya have been reported in countries found in the south of the European continent and in some southern states of USA (San Martín et al., 2010; Fredericks and Fernandez-Sesma, 2014; Amraoui and Failloux, 2016; Jourdain et al., 2020; Palermo et al., 2021; Fournet et al., 2023; Manica et al., 2023; Laverdeur et al., 2024; Rezza, 2024). Also, newly emerging arboviruses in previous endemic areas for these 3 common arboviruses have been detailed and studied by others (Wilder-Smith et al., 2017; Pérez et al., 2019; Caicedo et al., 2021; Wesselmann et al., 2024). Because of the unspecific flu-like symptoms caused by many of these viruses and the uncommon severe presentations of these infections, we might have been misdiagnosing some of these viral infections (Malavige et al., 2023; Mejía et al., 2023) or even worst, we have been missing novel virus discovery opportunities. It is tempting to speculate, that due to climate change, this will become an issue in many other geographical areas and not only in current hyper-endemic areas. Moreover, after the emergence of SARS-CoV-2, virus discovery for pandemic preparedness and advances in technologies such as novel diagnostic tools, treatments and vaccinations are required.

Tackling, preventing, and responding to arbovirus outbreaks involves various public health agencies and urges a comprehensive and collaborative approach, particularly but not only at the national level. A constant scarcity of resources in many of these hyperendemic regions such as Latin America, lack of multi-pathogen diagnostic kits for early detection, insufficiently trained clinical and vector control staff, and limited community awareness, continue as significant barriers to an efficient response. In this study, we highlight this issue in the studied area and suggest: i) better case surveillance with early detection and identification by non-stop and up-to-date training programs of the clinical staff for appropriate intervention and follow-up (if needed), ii) strengthening primary healthcare infrastructure for initial diagnosis, iii) community education and awareness programs for successful entomological surveillance, detection of potential breeding mosquito sites and their elimination, iv) personal protective measures such as usage of topical repellents or insecticides though community-wide must be recommended, v) an effective integrative vector control management approach should be implemented and innovative chemical, biological and/or genetic approaches must be developed and thus used, and vi) development of a low cost and effective vaccine against all arboviruses, and upon availability and approval, prioritization for these hyperendemic areas, together with advocacy efforts necessary to address vaccine hesitancy. These suggestions are not exhaustive. Furthermore, to effectively reduce outbreaks of these viruses, it is necessary that endemic nations establish infrastructure capable of facilitating comprehensive, cross-sectoral prevention and control initiatives. These programs should be jointly supported by local, state, and national government bodies (including health, environment, and education ministries), private companies, and most importantly, include community involvement.

The rise in both incidence and geographical reach of mosquito-transmitted viruses poses a significant challenge for healthcare systems across tropical and subtropical regions globally. Given the simultaneous widespread circulation of several arboviruses in these areas and due to shared epidemiological patterns and similar early-stage symptoms, their management is highly complex. Our study underscores the necessity for healthcare approaches in arbovirus management that encompass comprehensive molecular and serological diagnostic methods, alongside with genomic characterization. This broad strategy is crucial for achieving a more thorough comprehension of the clinical and epidemiological situation during arboviral disease outbreaks. Such insights can provide valuable direction for crafting and executing public health interventions and enhancing epidemiological surveillance measures.

## Data availability statement

The datasets presented in this study can be found in online repositories. The names of the repository/repositories and accession number(s) can be found in the article/**Supplementary Material**.

## Ethics statement

The studies involving humans were approved by Ethics Committee of the University of Costa Rica. The studies were conducted in accordance with the local legislation and institutional requirements. The ethics committee/institutional review board waived the requirement of written informed consent for participation from the participants or the participants’ legal guardians/next of kin because samples were send anonymously. No DNA or any information of the identity of the patients was obtained (and needed). Ethical approval was not required for the studies on animals in accordance with the local legislation and institutional requirements because only commercially available established cell lines were used.

## Author contributions

CS-G: Conceptualization, Data curation, Formal analysis, Investigation, Methodology, Validation, Visualization, Writing – original draft, Writing – review & editing. TM: Data curation, Formal analysis, Investigation, Methodology, Validation, Writing – original draft, Writing – review & editing. IC-P: Conceptualization, Data curation, Methodology, Resources, Writing – review & editing. JC-Á: Conceptualization, Formal analysis, Investigation, Methodology, Resources, Writing – review & editing. GP-H: Conceptualization, Methodology, Resources, Writing – review & editing. JD: Funding acquisition, Investigation, Methodology, Resources, Supervision, Writing – review & editing. AM-S: Data curation, Formal analysis, Funding acquisition, Investigation, Methodology, Resources, Validation, Writing – review & editing. EC-A: Conceptualization, Data curation, Formal analysis, Funding acquisition, Investigation, Methodology, Project administration, Resources, Supervision, Validation, Writing – original draft, Writing – review & editing.
