# Supplementary material for: Epidemiological, virological and clinical characterization of a Dengue/Zika outbreak in the Caribbean region of Costa Rica 2017–2018
Source: Front Cell Infect Microbiol. 2024 Jun 26;14:1421744. doi: 10.3389/fcimb.2024.1421744 (PMC11233455; doi:10.3389/fcimb.2024.1421744)
Supplement: Supplementary file 1 [file Table_1.docx]

| **Isolate name** | **Accession number** |
| --- | --- |
| DENV-2/CR/HC-08/2017 | PP702059 |
| DENV-2/CR/SQ-02/2017 | PP702060 |
| DENV-2/CR/SQ-10/2017 | PP702061 |
| DENV-2/CR/SQ-017/2017 | PP702062 |
| DENV-2/CR/SQ-21/2017 | PP702063 |
| DENV-2/CR/SQ-27/2017 | PP702064 |
| DENV-2/CR/SQ-57/2017 | PP702065 |
| DENV-2/CR/SQ-67/2017 | PP702066 |
| DENV-2/CR/SQ-120/2017 | PP702067 |
| DENV-2/CR/SQ-121/2017 | PP702068 |
| DENV-2/CR/SQ-146/2017 | PP702069 |
| DENV-2/CR/SQ-152/2017 | PP702070 |
| DENV-2/CR/SQ-170/2017 | PP702071 |
| DENV-2/CR/SQ-203/2017 | PP702072 |
| DENV-2/CR/SQ-268/2017 | PP702073 |
| DENV-2/CR/SQ-282/2017 | PP702074 |
| ZIKV/CR/SQ-077/2017 | PP708564 |

Suplementary table 1. Accession number of the sequences used for phylogenetic analysis.
